# Supplementary material for: Ecological assessment of water quality in freshwater wetlands based on the effect of environmental heterogeneity on phytoplankton communities in Northeast China
Source: PLoS One. 2024 Jul 8;19(7):e0306321. doi: 10.1371/journal.pone.0306321 (PMC11230543; doi:10.1371/journal.pone.0306321)
Supplement: S1 Table — (PDF) [file pone.0306321.s001.pdf]

S1 Table. *TSI(BOD)*, *TSI(COD)*, *TSI(TN)*, *TSI(TP)* in different sampling sites

		TSI(BOD)			*TSI (COD)*			*TSI (TN)*			*TSI (TP)*		
Year	Sampling sites												
2020	HLS1	42.640	47.856	40.594	79.581	83.583	82.607	76.921	84.014	70.052	40.594	82.607	70.052
2021	HLS1	43.124	47.856	41.123	78.732	83.403	82.731	73.421	81.278	73.421	41.123	82.731	73.421
2020	HLS2	35.839	43.124	36.491	77.329	79.651	77.405	74.234	81.452	66.272	36.491	77.405	66.272
2021	HLS2	36.491	44.062	37.125	76.403	79.651	78.003	70.052	77.801	67.886	37.125	78.003	67.886
2020	HLS3	37.125	43.598	37.742	76.793	79.651	76.870	73.967	81.624	66.272	37.742	76.870	66.272
2021	HLS3	36.491	43.598	37.125	76.638	79.442	77.631	69.710	78.224	67.497	37.125	77.631	67.497
2020	HLS4	35.839	44.517	36.491	79.160	79.511	76.716	74.755	81.794	66.272	36.491	76.716	66.272
2021	HLS4	35.839	44.062	36.491	78.947	80.806	79.371	69.360	77.366	69.004	36.491	79.371	69.004
2020	HLS5	43.598	48.991	43.124	79.927	83.703	82.105	78.014	84.454	71.356	43.124	82.105	71.356
2021	HLS5	43.598	47.856	42.640	80.064	80.806	82.731	75.752	81.794	75.752	42.640	82.731	75.752
2020	HLS6	37.125	43.598	37.125	77.101	79.581	76.870	74.755	81.452	65.403	37.125	76.870	65.403
2021	HLS6	37.125	43.124	37.125	76.716	78.150	77.329	69.710	77.145	69.360	37.125	77.329	69.360
2020	HLS7	36.491	44.062	37.742	77.253	80.472	76.793	75.010	81.794	65.843	37.742	76.793	65.843
2021	HLS7	36.491	42.640	38.926	76.870	79.301	78.515	69.710	75.261	75.992	38.926	78.515	75.992
2020	HLS8	42.146	47.856	41.640	80.269	83.703	82.420	77.145	84.161	70.387	41.640	82.420	70.387
2021	HLS8	42.146	46.661	42.146	79.720	82.105	82.105	73.967	80.746	73.421	42.146	82.105	73.421
