# Supplementary material for: Ecological assessment of water quality in freshwater wetlands based on the effect of environmental heterogeneity on phytoplankton communities in Northeast China
Source: PLoS One. 2024 Jul 8;19(7):e0306321. doi: 10.1371/journal.pone.0306321 (PMC11230543; doi:10.1371/journal.pone.0306321)
Supplement: S2 Table — (PDF) [file pone.0306321.s002.pdf]

S2 Table. Phytoplankton assemblages used for Redundancy analysis (RDA) (%)

| S | *An-ac* | *An-fa* | *An-fa-mi* | *An-sp* | *Co-de* | *Co-ob* | *Co-qu* | *Co-su* | *Ne-lu* | *Pe-te* | *Pe-te-te* | *Sc-bi* | *Sc-qu* | *St-am* | *We-bo* | *Di-so* | *An-os* | *Eu-al* | *Me-te* | *Os-pr* | *Os-te* | *Op-pa* | *Tr-mi* | *Ac-la* | *Ac-mi-sc* | *Ac-lat* |
|---|---|---|---|---|---|---|---|---|---|---|---|---|---|---|---|---|---|---|---|---|---|---|---|---|---|---|
| 1 | 0.38 | 0.37 | 0.25 | 0 | 0 | 5.57 | 0 | 1.44 | 1.37 | 0 | 0 | 0 | 0 | 0.35 | 0 | 3.08 | 0.44 | 2.9 | 2.78 | 0 | 0 | 0 | 1.47 | 0 | 0.49 | 7.82 |
| 2 | 0.25 | 0.33 | 2.09 | 0 | 0 | 1.07 | 0 | 1.06 | 0 | 0 | 0 | 0 | 0.76 | 1.78 | 0.91 | 1.5 | 2.09 | 1.1 | 3.21 | 0 | 0 | 1.08 | 1.57 | 0 | 4.46 | 3.72 |
| 3 | 2.1 | 7.89 | 1.01 | 3.01 | 0.13 | 0.33 | 0 | 0 | 0 | 1.2 | 0.89 | 1.27 | 6.21 | 0 | 2.08 | 1.67 | 1.07 | 0.78 | 0.79 | 0 | 0 | 1.83 | 0.91 | 1.81 | 1.25 | 6.09 |
| 4 | 3.09 | 2.11 | 4.09 | 8.09 | 0.61 | 0.66 | 0 | 0 | 0 | 1.71 | 0.39 | 3.07 | 7.41 | 0 | 5.31 | 1.3 | 2.71 | 1.05 | 0.64 | 1.71 | 1.21 | 2.41 | 2.9 | 3.93 | 1.67 | 3.12 |
| 5 | 0.58 | 0.86 | 1.07 | 1.33 | 1.05 | 1.01 | 0.54 | 1.47 | 0 | 0 | 0 | 0.33 | 0.64 | 0 | 0.43 | 4.51 | 3.08 | 1.51 | 2.29 | 0.89 | 1.28 | 2.19 | 2.8 | 1.1 | 3.83 | 1.35 |
| 6 | 2.31 | 1.25 | 1.59 | 1.2 | 3.21 | 4.56 | 2.13 | 0.79 | 3.17 | 1.35 | 1.32 | 1.23 | 1.21 | 1.31 | 0 | 1.57 | 2.18 | 1.31 | 0 | 1.53 | 0.96 | 4.62 | 3.51 | 0 | 5.56 | 0 |
| 7 | 0.78 | 0.96 | 1.21 | 0 | 0.51 | 0.37 | 0 | 0.56 | 0.59 | 0 | 0.21 | 0.21 | 1.3 | 0.58 | 0 | 7.58 | 4.58 | 0.98 | 0.33 | 0 | 0 | 1.26 | 3.02 | 0.31 | 3.21 | 2.35 |
| 8 | 0.53 | 0.57 | 1.35 | 0 | 0.31 | 0 | 0 | 0.31 | 0.45 | 0 | 0.35 | 0.32 | 1.5 | 0.97 | 0 | 6.43 | 2.31 | 1.2 | 0.39 | 0 | 0 | 0 | 0 | 0.25 | 4.25 | 3.21 |
| 9 | 1.2 | 3.21 | 2.13 | 0 | 0 | 0 | 0 | 0 | 0 | 0 | 0.65 | 0.89 | 1.8 | 0 | 0 | 0 | 3.25 | 0 | 0.21 | 0 | 0 | 0 | 0 | 0.33 | 30.28 | 4.35 |
| 10 | 2.17 | 2.59 | 2.09 | 0 | 0 | 0 | 0 | 0 | 0 | 0 | 0.68 | 0.67 | 1.89 | 0 | 0 | 0 | 4.65 | 0 | 0.18 | 0 | 0 | 0 | 0 | 0.67 | 26.21 | 2.14 |
| 11 | 0.97 | 1.05 | 1.65 | 0 | 0.31 | 0 | 0 | 0.39 | 0.32 | 0 | 0.34 | 0.33 | 0.27 | 0 | 0 | 3.21 | 2.31 | 1.35 | 0.29 | 0 | 0 | 1.52 | 3.21 | 0.78 | 1.56 | 1.23 |
| 12 | 0.57 | 0.65 | 1.89 | 0 | 0.54 | 0.86 | 0 | 0.38 | 0.65 | 0 | 0.35 | 0.19 | 1.35 | 0.51 | 0 | 2.31 | 1.09 | 1.2 | 0.21 | 0 | 0 | 1.78 | 2.13 | 0.26 | 2.07 | 1.08 |
| 13 | 0.91 | 1.71 | 1.4 | 1.53 | 0 | 0 | 0 | 0 | 0 | 2.03 | 1.25 | 3.25 | 4.65 | 0 | 0 | 0 | 0 | 0.31 | 0.53 | 7.32 | 10.25 | 0 | 0 | 0 | 0 | 0 |
| 14 | 0.76 | 0.81 | 1.23 | 2.37 | 0 | 0 | 0 | 0 | 0 | 1.07 | 1.04 | 4.12 | 5.79 | 0 | 0 | 0 | 0 | 0.21 | 1.13 | 5.93 | 12.32 | 0 | 0 | 0 | 0 | 0 |
| 15 | 1.21 | 3.05 | 0.89 | 2.79 | 0 | 0 | 0 | 0 | 0 | 3.05 | 1.76 | 2.3 | 0.71 | 0 | 0 | 0 | 0 | 1.21 | 1.89 | 4.23 | 8.65 | 0 | 0 | 0 | 0 | 0 |
| 16 | 2.12 | 2.13 | 0.75 | 1.58 | 0 | 0 | 0 | 0 | 0 | 1.82 | 1.95 | 3.71 | 0.39 | 0 | 0 | 0 | 0 | 2.2 | 1.57 | 1.08 | 10.52 | 0 | 0 | 0 | 0 | 0 |
| 17 | 1.11 | 1.35 | 0.87 | 2.51 | 0 | 0 | 0 | 0 | 0 | 0.76 | 1.82 | 3.51 | 1.26 | 0 | 0 | 0 | 0 | 0.68 | 0.75 | 2.14 | 23.45 | 0 | 0 | 0 | 0 | 0 |
| 18 | 1.21 | 1.53 | 1.25 | 2.31 | 0 | 0 | 0 | 0 | 0 | 1.03 | 2.11 | 2.31 | 0.98 | 0 | 0 | 0 | 0 | 0.13 | 0.15 | 1.97 | 7.82 | 0 | 0 | 0 | 0 | 0 |
| 19 | 0.52 | 0.98 | 1.32 | 0.78 | 0.76 | 0.96 | 0 | 0.21 | 0.63 | 0 | 0.13 | 0.71 | 1.26 | 0 | 0 | 2.35 | 6.57 | 0.27 | 0.27 | 0.18 | 1.08 | 0 | 1.05 | 0.29 | 2.26 | 0 |
| 20 | 0.59 | 0.67 | 1.45 | 0.59 | 0.83 | 0.52 | 0 | 0.35 | 0.5 | 0 | 0.19 | 0.45 | 1.37 | 0 | 0 | 1.99 | 4.82 | 0.19 | 0.51 | 0.23 | 2.13 | 0 | 1.21 | 0.58 | 4.53 | 1.37 |
| 21 | 0.79 | 1.25 | 2.53 | 1.21 | 0 | 0 | 0 | 0 | 0 | 1.63 | 0.51 | 1.01 | 1.52 | 0 | 0 | 0 | 3.19 | 0 | 0.33 | 0.79 | 3.1 | 0 | 0.78 | 0.71 | 1.89 | 1.45 |
| 22 | 0.67 | 2.03 | 2.78 | 1.07 | 0 | 0 | 0 | 0 | 0 | 1.81 | 0.52 | 0.97 | 1.01 | 0 | 0 | 0 | 4.06 | 0 | 0.22 | 0.85 | 4.12 | 0 | 0.56 | 0.62 | 1.82 | 2.57 |
| 23 | 0.21 | 1.21 | 1.61 | 0.83 | 0.57 | 0.72 | 0 | 0.19 | 0.48 | 0 | 0.37 | 0.62 | 0.98 | 0 | 0 | 2.31 | 2.79 | 0.73 | 0.27 | 0.71 | 2.2 | 0 | 2.31 | 0.53 | 2.32 | 2.19 |
| 24 | 0.51 | 1.07 | 1.35 | 0.52 | 0.54 | 0.77 | 0 | 0.37 | 0.57 | 1.05 | 0.28 | 0.75 | 1.32 | 0 | 0 | 5.45 | 3.51 | 0.82 | 0.3 | 0.82 | 1.31 | 0 | 1.98 | 0.58 | 1.36 | 2.39 |
| 25 | 0 | 0 | 0 | 0 | 1.17 | 3.39 | 3.09 | 5.38 | 2.76 | 0 | 0 | 0 | 0 | 3.51 | 0 | 7.32 | 0.98 | 2.17 | 0 | 0.97 | 0.21 | 4.53 | 1.34 | 0 | 0.31 | 0 |
| 26 | 0 | 0 | 0 | 0 | 5.52 | 17.69 | 2.9 | 7.92 | 0 | 0.41 | 0.27 | 0 | 0 | 2.31 | 0 | 2.09 | 0 | 0 | 0 | 3.67 | 0 | 3.21 | 0 | 0 | 0.25 | 0 |

| | | | | | | | | | | | | | | | | | | | | | | | | | | |
|---|---|---|---|---|---|---|---|---|---|---|---|---|---|---|---|---|---|---|---|---|---|---|---|---|---|---|
| 27 | 3.37 | 5.34 | 12.76 | 15.32 | 0.28 | 1.69 | 0 | 0 | 0 | 2.53 | 3.09 | 0 | 0 | 3.51 | 8.76 | 0.31 | 0.76 | 0 | 0 | 0.26 | 0 | 0 | 0 | 0 | 2.75 | 0 |
| 28 | 3.56 | 6.38 | 9.57 | 14.2 | 0 | 2.07 | 0 | 0 | 0 | 1.33 | 2.37 | 0 | 0 | 4.53 | 7.65 | 0.26 | 0.98 | 0 | 0.78 | 0.98 | 0 | 0 | 0 | 0 | 3.28 | 0 |
| 29 | 0.78 | 1.36 | 0.31 | 1.67 | 1.08 | 1.12 | 0.86 | 1.28 | 0.27 | 0 | 0 | 0 | 0 | 0 | 0 | 1.51 | 0.52 | 2.13 | 0.75 | 0.69 | 0.57 | 1.32 | 0.71 | 0 | 0 | 0 |
| 30 | 0 | 0.37 | 0 | 0 | 3.52 | 1.81 | 0.39 | 1.25 | 5.79 | 0 | 0 | 0 | 0 | 0 | 0 | 1.45 | 2.1 | 3.01 | 0 | 0.78 | 0.33 | 1.87 | 0 | 0 | 0 | 0 |
| 31 | 0 | 0 | 0 | 0 | 1.17 | 2.39 | 1.09 | 5.38 | 2.76 | 0 | 0 | 0 | 0 | 3.51 | 0 | 7.32 | 0.98 | 2.17 | 0 | 0.97 | 0.21 | 4.53 | 1.34 | 1.39 | 1.17 | 0 |
| 32 | 0 | 0 | 0 | 0 | 5.52 | 10.69 | 0.98 | 4.92 | 0 | 0.41 | 0.27 | 0 | 0 | 2.31 | 0 | 2.09 | 0 | 0 | 0 | 3.67 | 0 | 3.21 | 0 | 2.39 | 0.98 | 0 |
| 33 | 1.37 | 14.32 | 1.76 | 1.32 | 0.28 | 3.69 | 0 | 0 | 0 | 2.53 | 3.09 | 3.46 | 6.79 | 3.51 | 8.76 | 0.31 | 0.76 | 0 | 0 | 0.26 | 0 | 0 | 0 | 0.81 | 3.79 | 0 |
| 34 | 2.65 | 12.38 | 0.57 | 2.89 | 0 | 2.07 | 0 | 0 | 0 | 1.33 | 2.37 | 1.65 | 9.85 | 4.53 | 7.65 | 0.26 | 0.98 | 0 | 0.28 | 0.98 | 0 | 0 | 0 | 1.25 | 2.38 | 0 |
| 35 | 0.78 | 1.36 | 0.31 | 5.67 | 11.08 | 3.1 | 0.76 | 2.28 | 0.31 | 0 | 0 | 0 | 0.27 | 1.26 | 0 | 2.51 | 0.52 | 2.13 | 0.75 | 0.69 | 0.57 | 1.32 | 0.71 | 0 | 0.59 | 3.64 |
| 36 | 0 | 0.26 | 0.38 | 0 | 1.05 | 2.37 | 0.39 | 1.25 | 1.79 | 0 | 0 | 0.21 | 0.39 | 1.32 | 0 | 2.35 | 0.79 | 0.21 | 0 | 1.01 | 0.29 | 0 | 0 | 0.27 | 1.33 | 2.31 |
| 37 | 0.27 | 0 | 0.45 | 0.23 | 0 | 3.17 | 0 | 2.31 | 2.08 | 0 | 0 | 0.53 | 0.31 | 2.57 | 0.76 | 2.98 | 0.41 | 1.95 | 1.76 | 0 | 0 | 0 | 1.56 | 0.87 | 0.49 | 5.47 |
| 38 | 0.45 | 0 | 1.87 | 0.19 | 0 | 2.15 | 0 | 2.57 | 2.76 | 0 | 0 | 0.47 | 0.45 | 2.33 | 0.89 | 1.72 | 0.33 | 1.86 | 1.21 | 0 | 0 | 0 | 2.31 | 0.65 | 0.31 | 3.96 |
| 39 | 2.28 | 3.52 | 1.73 | 2.12 | 0 | 0 | 0 | 0 | 0 | 0 | 0 | 0.98 | 0.82 | 0 | 2.31 | 1.53 | 0.29 | 0.77 | 0.97 | 0 | 0 | 0 | 1.21 | 1.53 | 2.51 | 4.21 |
| 40 | 2.09 | 2.79 | 2.31 | 3.07 | 0 | 0 | 0 | 0 | 0 | 0 | 0 | 0.78 | 1.2 | 0 | 3.57 | 1.31 | 0.25 | 0.52 | 1.1 | 0 | 0 | 0 | 2.76 | 2.47 | 2.31 | 3.33 |
| 41 | 1.03 | 0 | 1.63 | 1.21 | 2.33 | 1.97 | 0.78 | 0 | 0 | 0 | 0 | 0.99 | 0.27 | 1.79 | 0.86 | 2.41 | 0.36 | 1.27 | 1.62 | 0 | 0 | 0 | 2.35 | 1.21 | 2.82 | 2.76 |
| 42 | 1.51 | 0 | 2.32 | 0.98 | 3.15 | 2.52 | 1.31 | 0 | 0 | 0 | 0 | 0.54 | 0.66 | 3.21 | 0.29 | 2.03 | 0.21 | 0.78 | 1.51 | 0 | 0 | 0 | 2.91 | 1.31 | 3.56 | 1.92 |
| 43 | 0.51 | 0.78 | 1.32 | 0 | 1.31 | 0 | 0 | 0.37 | 0.97 | 0.29 | 0.37 | 0.21 | 0.31 | 2.54 | 0 | 3.58 | 2.12 | 0.86 | 0.76 | 0.21 | 0.23 | 0.81 | 2.03 | 0.35 | 2.51 | 0 |
| 44 | 0.49 | 0.83 | 1.75 | 0 | 0.97 | 0 | 0 | 0.29 | 0.86 | 0.71 | 0.45 | 0.17 | 0.16 | 1.66 | 0 | 4.62 | 3.15 | 1.02 | 0.89 | 0.75 | 0.57 | 0.52 | 3.21 | 0.45 | 2.17 | 1.52 |
| 45 | 1.35 | 2.19 | 2.31 | 0.27 | 0 | 0 | 0 | 0 | 0 | 0.34 | 0.57 | 0.39 | 0.41 | 0 | 0 | 0 | 4.23 | 0 | 1.03 | 0.93 | 0.82 | 0.76 | 0 | 0.27 | 4.32 | 2.31 |
| 46 | 1.97 | 2.31 | 1.89 | 0.35 | 0 | 0 | 0 | 0 | 0 | 0.51 | 0.62 | 0.76 | 0.28 | 0 | 0 | 0 | 5.17 | 0 | 0.77 | 0.44 | 0.95 | 0.37 | 0 | 0.65 | 5.71 | 4.21 |
| 47 | 0.32 | 1.45 | 1.75 | 0 | 0.9 | 0.25 | 0 | 0.71 | 0.52 | 0.79 | 0.93 | 0.71 | 0.54 | 0 | 0 | 4.35 | 3.39 | 1.27 | 1.25 | 0.19 | 0.76 | 0.21 | 0 | 0.92 | 3.76 | 3.19 |
| 48 | 0.97 | 0.66 | 2.05 | 0 | 0.65 | 0.31 | 0 | 0.39 | 0.75 | 0.91 | 1.07 | 0.53 | 0.42 | 0 | 0 | 5.12 | 2.76 | 1.19 | 0.91 | 0.25 | 0.79 | 0.65 | 0 | 0.53 | 4.11 | 2.51 |
| 49 | 0.82 | 1.2 | 1.25 | 0 | 0 | 0 | 0 | 0 | 0 | 1.06 | 0.94 | 3.12 | 2.71 | 0 | 0 | 0 | 0 | 0.97 | 0.53 | 19.15 | 10.76 | 0.21 | 0 | 0 | 0 | 0 |
| 50 | 0.98 | 1.52 | 1.17 | 2.15 | 0 | 0 | 0 | 0 | 0 | 2.01 | 1.52 | 1.96 | 2.31 | 0 | 0 | 0 | 0 | 0.69 | 0.97 | 16.21 | 10.89 | 0.32 | 0 | 0 | 0 | 0 |
| 51 | 1.35 | 2.95 | 0.69 | 2.12 | 0 | 0 | 0 | 0 | 0 | 3.15 | 1.61 | 2.31 | 1.96 | 0 | 0 | 0 | 0 | 1.51 | 1.52 | 3.76 | 8.76 | 0.41 | 0 | 0 | 0 | 0 |
| 52 | 2.97 | 1.99 | 0.35 | 1.21 | 0 | 0 | 0 | 0 | 0 | 1.62 | 1.87 | 2.47 | 1.54 | 0 | 0 | 0 | 0 | 1.17 | 1.36 | 4.21 | 9.65 | 0.76 | 0 | 0 | 0 | 0 |
| 53 | 1.26 | 1.42 | 0.97 | 2.35 | 0 | 0 | 0 | 0 | 0 | 1.31 | 1.91 | 1.51 | 1.28 | 0 | 0 | 0 | 0 | 0.52 | 1.02 | 3.23 | 5.47 | 0.53 | 0 | 0 | 0 | 0 |
| 54 | 1.02 | 1.31 | 1.45 | 1.82 | 0 | 0 | 0 | 0 | 0 | 1.56 | 0 | 1.91 | 1.71 | 0 | 0 | 0 | 0 | 0.49 | 0.89 | 2.97 | 7.93 | 0.32 | 0 | 0 | 0 | 0 |
| 55 | 1.43 | 2.05 | 1.47 | 2.66 | 0 | 0 | 0 | 2.31 | 0 | 0 | 0 | 2.71 | 2.82 | 0 | 0 | 0 | 3.26 | 0.28 | 1.42 | 2.15 | 2.98 | 0 | 0.25 | 0.41 | 2.33 | 0 |

| | | | | | | | | | | | | | | | | | | | | | | | | | | |
|---|---|---|---|---|---|---|---|---|---|---|---|---|---|---|---|---|---|---|---|---|---|---|---|---|---|---|
| 56 | 1.29 | 2.21 | 1.32 | 2.25 | 0 | 0 | 0 | 0 | 1.82 | 1.71 | 0 | 2.05 | 3.95 | 0 | 0 | 0 | 3.19 | 0.15 | 1.38 | 2.73 | 3.86 | 0 | 0.21 | 0.46 | 2.25 | 0 |
| 57 | 3.97 | 3.75 | 1.95 | 2.31 | 0 | 0 | 0 | 0 | 0 | 1.89 | 0 | 2.21 | 3.9 | 0 | 0 | 0 | 4.25 | 0.13 | 2.03 | 2.62 | 3.52 | 0 | 0.19 | 0.47 | 2.17 | 0 |
| 58 | 4.12 | 3.33 | 2.08 | 1.97 | 0 | 0 | 0 | 0 | 0 | 0 | 0 | 5.24 | 4.57 | 0 | 0 | 0 | 3.76 | 0.16 | 2.05 | 3.01 | 3.31 | 0 | 0.13 | 0.38 | 2.16 | 0 |
| 59 | 1.31 | 2.76 | 1.42 | 1.95 | 0 | 0 | 0 | 1.75 | 1.51 | 0 | 0 | 4.16 | 5.67 | 0 | 0 | 0 | 3.78 | 0.35 | 1.57 | 2.33 | 2.46 | 0 | 0 | 0.59 | 3.22 | 0 |
| 60 | 1.33 | 2.28 | 1.25 | 1.83 | 0 | 0 | 0 | 1.39 | 1.76 | 0 | 0 | 3.52 | 3.96 | 0 | 0 | 0 | 4.56 | 0.33 | 1.81 | 2.47 | 2.53 | 0 | 0 | 0.5 | 3.27 | 0 |
| 61 | 0 | 0 | 0 | 0 | 2.35 | 2.51 | 2.07 | 2.37 | 0 | 1.41 | 0 | 0 | 0 | 2.57 | 1.21 | 7.52 | 0.96 | 2.01 | 0.26 | 0 | 0 | 0 | 1.21 | 0 | 2.33 | 1.09 |
| 62 | 0 | 0 | 0 | 0 | 3.62 | 3.15 | 2.76 | 4.32 | 0 | 1.53 | 0.72 | 0 | 0 | 3.31 | 1.35 | 5.43 | 0.95 | 1.76 | 0.21 | 0 | 0 | 0 | 1.33 | 0 | 2.17 | 1.81 |
| 63 | 2.29 | 2.35 | 2.76 | 3.35 | 0 | 0 | 0 | 0 | 0 | 1.76 | 2.83 | 0 | 0 | 4.62 | 4.31 | 0 | 0.49 | 0.45 | 0.23 | 0 | 0 | 0 | 1.45 | 0 | 23.15 | 1.23 |
| 64 | 4.31 | 3.97 | 3.15 | 2.61 | 0 | 0 | 0 | 0 | 0 | 1.89 | 1.57 | 0 | 0 | 5.39 | 5.27 | 0 | 0.89 | 0.78 | 0.31 | 0 | 0 | 0 | 1.57 | 0 | 25.2 | 1.51 |
| 65 | 0.52 | 1.66 | 1.66 | 0 | 1.92 | 2.24 | 1.33 | 2.1 | 0.97 | 0 | 1.69 | 0 | 0 | 0 | 2.1 | 4.31 | 1.2 | 1.52 | 0.27 | 0 | 0 | 0 | 1.21 | 0 | 3.21 | 1.76 |
| 66 | 0 | 0.96 | 0.99 | 0 | 2.15 | 1.75 | 1.69 | 1.75 | 2.85 | 0 | 0 | 0 | 0 | 0 | 1.59 | 6.12 | 0.73 | 1.76 | 0.52 | 0 | 0 | 0 | 1.42 | 0 | 2.96 | 1.22 |
| 67 | 0 | 0 | 0 | 0 | 1.05 | 20.13 | 2.38 | 3.27 | 1.21 | 0.42 | 0 | 0 | 0 | 2.16 | 0 | 3.97 | 0.75 | 2.13 | 0 | 0.37 | 0 | 0 | 1.53 | 1.46 | 0.16 | 0.59 |
| 68 | 0 | 0 | 0 | 0 | 1.51 | 19.2 | 2.52 | 3.1 | 0 | 0.53 | 0.73 | 0 | 0 | 2.31 | 0 | 2.89 | 0.24 | 1.73 | 0 | 0.82 | 0 | 0 | 1.32 | 1.97 | 0.23 | 0.71 |
| 69 | 1.56 | 3.32 | 2.15 | 2.3 | 0.51 | 2.31 | 0 | 0 | 0 | 1.07 | 2.19 | 4.57 | 3.19 | 2.87 | 0 | 1.31 | 0.29 | 0.76 | 0 | 0.41 | 0 | 0 | 1.07 | 1.88 | 0.34 | 0.87 |
| 70 | 2.61 | 2.56 | 2.58 | 1.96 | 0.21 | 3.09 | 0 | 0 | 0 | 1.22 | 1.98 | 2.63 | 4.58 | 2.95 | 0 | 1.07 | 0.72 | 0.82 | 0 | 0.33 | 0 | 0 | 0.98 | 1.66 | 0.57 | 0.33 |
| 71 | 1.07 | 0.98 | 0.91 | 3.61 | 1.32 | 3.31 | 0 | 0.85 | 0.15 | 0.96 | 0 | 1.63 | 2.72 | 1.25 | 0 | 2.44 | 0.89 | 1.53 | 0 | 0.27 | 0 | 0 | 1.13 | 1.09 | 1.21 | 0.29 |
| 72 | 0.39 | 0 | 0.62 | 0 | 1.71 | 2.2 | 0 | 2.31 | 1.82 | 1.13 | 0 | 1.39 | 2.33 | 1.26 | 0 | 2.15 | 0.25 | 1.42 | 0 | 0.66 | 0 | 0 | 2.22 | 1.52 | 1.33 | 0.95 |
| 73 | 0 | 0 | 0.544872 | 0.019231 | 0 | 0.121795 | 0.019231 | 0 | 0.012821 | 0 | 0.23 | 0 | 0.19 | 0.19 | 0.02 | 0 | 0 | 0 | 0 | 0.16 | 0.2 | 0 | 0 | 0 | 0 | 6.96 |
| 74 | 0 | 0 | 0 | 0.08 | 0 | | | | | | 0.12 | 0.032 | 0 | 0.087 | 0.33 | 0.23 | 0.12 | 0.087 | 0.23 | 0 | 0 | 0 | 0 | 0 | 0 | 7.33 |
| 75 | 0 | 0 | 0 | 0.12 | 0 | 0 | 0 | 0 | 0 | 0 | 0.14 | 0 | 0 | 0 | 0 | 0 | 0 | 0 | 0.04 | 0.12 | 0 | 0 | 0 | 0 | 0 | 8.12 |
| 76 | 0.04 | 0.03 | 0.08 | 0.06 | 0.02 | 0.1 | 0.05 | 0.03 | 0.08 | 0.34 | 0.09 | | 0.04 | 0.27 | 0.03 | 0.03 | 0.012 | 0.09 | 0.02 | 0.11 | 0 | 0 | 0 | 0 | 0 | 5.16 |
| 77 | 0.05 | 0.05 | 0.36 | 0.16 | 0.03 | 0.17 | 0.06 | | 0.06 | 0.23 | 0.32 | 0.34 | | 0.05 | | 0.03 | | 0.09 | 0.21 | 0.05 | 0.36 | 0 | 0 | 0 | 0 | 1.21 |
| 78 | 0 | 0 | 0 | 0.21 | 0 | 0.17 | 0.34 | 0.13 | 0.09 | 0.32 | 0 | 0 | 0.23 | 0.11 | 0 | 0 | 0 | 0 | 0.21 | 0 | 0 | 0 | 0 | 0 | 0 | 1.33 |
| 79 | 0.23 | 0.13 | 0.32 | 0.09 | 0.23 | 0 | 0 | 0 | 0 | 0 | 0.11 | 0 | 0 | 0.04 | 0 | 0.11 | 0.11 | 0.07 | 0 | 0 | 0 | 0.41 | 2.33 | 0 | 0 | 1.77 |
| 80 | 0.21 | 0.23 | 0.13 | 0.32 | 0.09 | 0.23 | 0 | 0 | 0 | 0 | 0 | 0.11 | 0 | 0 | 0.04 | 0 | 0.11 | 0.11 | 0.07 | 0 | 0 | 0.46 | 2.25 | 0 | 0 | 1.72 |
| 81 | 0.53 | 0 | 0.33 | 0.21 | 0 | 0 | 0 | 0 | 0 | 0 | 0 | 0 | 0 | 0 | 0 | 0.031746 | 0.31 | 0 | 0 | 0 | 0 | 0 | 0 | 0 | 0 | 0 |
| 82 | 0.47 | 0 | 0 | 0.21 | | 0.065217 | 0 | 0 | 0 | 0 | 0.11 | 0.21 | 0.21 | 0.24 | 0.21 | 0.11 | 0 | 0 | 0 | | 0 | 0 | 0 | 0 | 0 | 0 |
| 83 | 0 | 0 | 0 | 0 | 2.35 | 2.51 | 2.07 | 2.37 | 0 | 1.41 | 0 | 0 | 0 | 2.57 | 1.21 | 7.52 | 0.96 | 2.01 | 0.26 | 0 | 0 | 0 | 0 | 0.21 | 0.19 | 0.087 |
| 84 | 0 | 0 | 0 | 0 | 3.62 | 3.15 | 2.76 | 4.32 | 0 | 1.53 | 0.72 | 0 | 0 | 3.31 | 1.35 | 5.43 | 0.95 | 1.76 | 0.21 | 0 | 0 | 0 | 1.33 | 0 | 2.17 | 1.81 |

| | | | | | | | | | | | | | | | | | | | | | | | | | | |
|---|---|---|---|---|---|---|---|---|---|---|---|---|---|---|---|---|---|---|---|---|---|---|---|---|---|---|
| 85 | 2.29 | 2.35 | 2.76 | 3.35 | 0 | 0 | 0 | 0 | 0 | 1.76 | 2.83 | 0 | 0 | 4.62 | 4.31 | 0 | 0.49 | 0.45 | 0.23 | 0 | 0 | 0 | 1.45 | 0 | 23.15 | 1.23 |
| 86 | 4.31 | 3.97 | 3.15 | 2.61 | 0 | 0 | 0 | 0 | 0 | 1.89 | 1.57 | 0 | 0 | 5.39 | 5.27 | 0 | 0.89 | 0.78 | 0.31 | 0 | 0 | 0 | 1.57 | 0 | 25.2 | 1.51 |
| 87 | 0.52 | 1.66 | 1.66 | 0 | 1.92 | 2.24 | 1.33 | 2.1 | 0.97 | 0 | 1.69 | 0 | 0 | 0 | 2.1 | 4.31 | 1.2 | 1.52 | 0.27 | 0 | 0 | 0 | 1.21 | 0 | 3.21 | 1.76 |
| 88 | 0 | 0.96 | 0.99 | 0 | 2.15 | 1.75 | 1.69 | 1.75 | 2.85 | 0 | 0 | 0 | 0 | 0 | 1.59 | 6.12 | 0.73 | 1.76 | 0.52 | 0 | 0 | 0 | 1.42 | 0 | 2.96 | 1.22 |
| 89 | 0 | 0 | 0 | 0 | 0 | 0 | 0.51 | 0 | 0 | 0.67 | 0 | 0 | 0 | 0 | 0 | 0 | 0 | 0.51 | 0 | 0 | 0.67 | 0 | 1.53 | 1.46 | 0.16 | 0.59 |
| 90 | 0.82 | 0.97 | 1.21 | 0.59 | 1.13 | 0 | 0.43 | 0 | 0 | 0 | 0.23 | 0.82 | 0.97 | 1.21 | 0.59 | 1.13 | 0 | 0.43 | 0 | 0 | 0 | 0.23 | 1.32 | 1.97 | 0.23 | 0.71 |
| 91 | 0 | 0 | 0 | 0 | 0 | 0 | 0.71 | 0.43 | 0 | 0 | 0.67 | 0 | 0 | 0 | 0 | 0 | 0 | 0.71 | 0.43 | 0 | 0 | 0.67 | 0.19 | 0.37 | 0.46 | 0.36 |
| 92 | 0.29 | 0.71 | 0.29 | 0.51 | 0.43 | 0 | 0.29 | 0.33 | 0 | 0 | 1.07 | 0.29 | 0.71 | 0.29 | 0.51 | 0.43 | 0 | 0.29 | 0.33 | 0 | 0 | 1.07 | 0 | 0 | 0 | 0 |
| 93 | 0.51 | 0.43 | 0.33 | 0 | 0 | 0 | 0.51 | 0 | 0 | 0.23 | 0 | 0.51 | 0.43 | 0.33 | 0 | 0 | 0 | 0.51 | 0 | 0 | 0.23 | 0 | 0 | 0 | 0 | 0 |
| 94 | 0 | 0 | 0 | 0 | 0 | 0 | 0 | 0 | 0 | 0 | 0 | 0 | 0 | 0 | 0 | 0 | 0 | 0 | 0 | 0 | 0 | 0 | 0 | 0 | 0 | 0.21 |
| 95 | 0 | 0 | 0 | 0.23 | 0.67 | 0.59 | 0 | 0.51 | 0 | 0 | 0.23 | 0 | 0 | 0 | 0.23 | 0.67 | 0.59 | 0 | 0.51 | 0 | 0 | 0.23 | 1.72 | 0.85 | 0.73 | 0 |
| 96 | 1.28 | 1.31 | 1.07 | 0 | 0 | 1.13 | 0 | 0.43 | 0 | 0 | 0.67 | 1.28 | 1.31 | 1.07 | 0 | 0 | 1.13 | 0 | 0.43 | 0 | 0 | 0.67 | 0 | 0 | 0 | 0.31 |
