# Supplementary figures and images for: Ecological assessment of water quality in freshwater wetlands based on the effect of environmental heterogeneity on phytoplankton communities in Northeast China
Source: PLoS One. 2024 Jul 8;19(7):e0306321. doi: 10.1371/journal.pone.0306321 (PMC11230543; doi:10.1371/journal.pone.0306321)

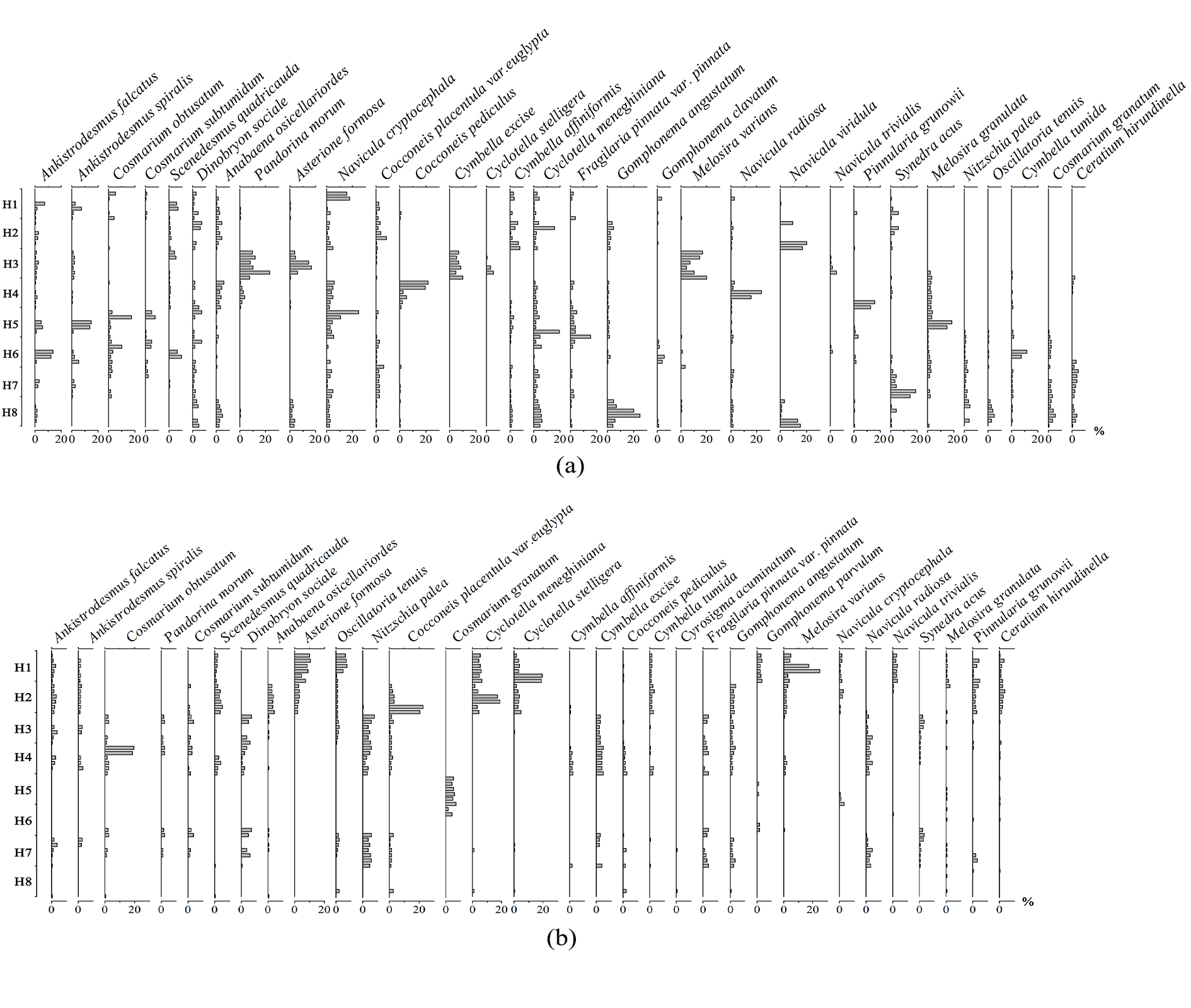

Supplement: S1 Fig — Assemblage composition and relative abundance of the most common species in eight sites (a. abundance of the species in 2020; b. abundance of the species in 2021). (TIF) [file pone.0306321.s003.tif]

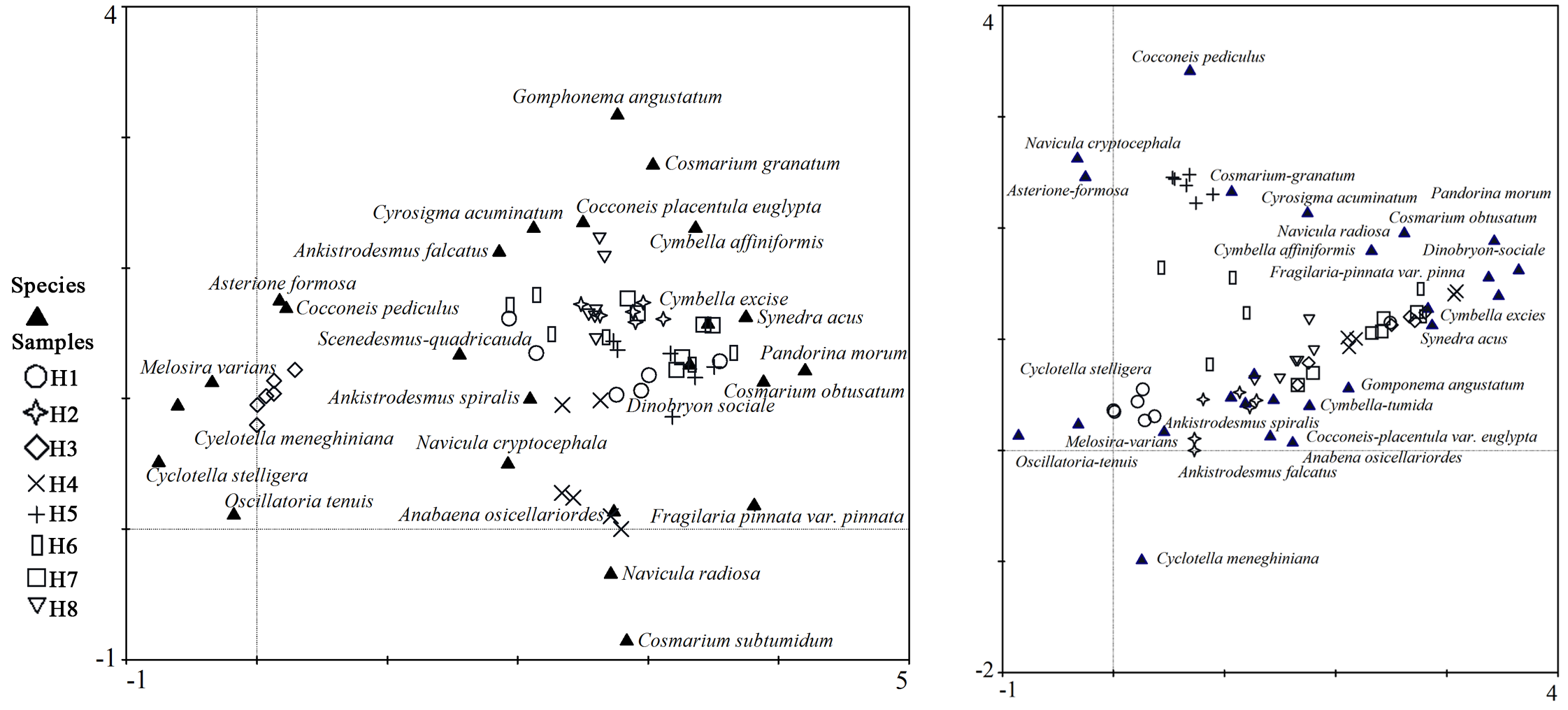

Supplement: S2 Fig — Detrended correspondence analysis (DCA) of algae assemblages in different sites during two years (a. DCA in 2020; b. DCA in 2021). (TIF) [file pone.0306321.s004.tif]
