# Supplementary material for: Ecological assessment of water quality in freshwater wetlands based on the effect of environmental heterogeneity on phytoplankton communities in Northeast China
Source: PLoS One. 2024 Jul 8;19(7):e0306321. doi: 10.1371/journal.pone.0306321 (PMC11230543; doi:10.1371/journal.pone.0306321)
Supplement: Attachment — Submitted filename: Review of Manuscript by Dr. Adzigbli.pdf [file pone.0306321.s006.pdf]

Ecological Assessment of Water Quality in Freshwater Wetlands: Effect of Environmental Heterogeneity on Phytoplankton Communities in Northeast China

Abstract:

The study delves into how phytoplankton diversity can act as a mirror for water quality within the Hulanhe Wetland. It pinpoints nutrient levels (TN and TP) and temperature as pivotal environmental factors shaping the phytoplankton community's structure. This work holds significance for aquatic ecology and environmental assessment, offering insights crucial for the efficient monitoring and management of wetland ecosystems.

Introduction:

The introduction lays the groundwork by highlighting why understanding phytoplankton diversity matters in assessing water quality in freshwater wetlands. It also discusses existing research on using phytoplankton as indicators of water quality and the various factors influencing phytoplankton communities.

Methods:

The methods section meticulously describes the sampling and analysis procedures, covering everything from water quality measurements to phytoplankton identification, enumeration, and diversity index calculations. The authors followed standard protocols, ensuring a robust and replicable methodology.

Results:

The results section presents the study's findings, particularly focusing on how nutrient levels and temperature affect phytoplankton communities in the Hulanhe Wetland. It emphasizes the link between environmental variables and phytoplankton diversity.

Discussion:

In the discussion, the results are put into context within the existing body of knowledge. The implications of the findings for understanding water quality in freshwater wetlands are explored, with an eye towards improving wetland ecosystem monitoring and management strategies.

Conclusion:

The conclusion summarizes the main findings regarding how environmental factors influence phytoplankton diversity. It underscores the study's importance for advancing knowledge in aquatic ecology and environmental assessment.

Recommendation:

I would encourage the authors to submit a revised version of their manuscript, as their work shows promise in contributing to the field. Areas for improvement could include addressing data deposition in repositories, adhering to relevant guidelines, making the manuscript more accessible to non-specialists, and clarifying the availability of any software created during the study. Additionally, assessing whether the manuscript contains any experiments of Dual Use concern, as defined by NIH, would be beneficial.
